# Title: efficacy of intravitreal dexamethasone implant on hard exudate in diabetic macular edema

**DOI:** 10.1186/s12886-020-01786-2

**Published:** 2021-01-15

**Authors:** Chang Ki Yoon, Min Sagong, Jae Pil Shin, Sang Joon Lee, Joo Eun Lee, Ji Eun Lee, Inyoung Chung, Woo Jin Jeong, Kang Yeun Pak, Hyun Woong Kim

**Affiliations:** 1 Department of Ophthalmology, Seoul National University Hospital, Seoul, Korea; 2 Department of Ophthalmology, Yeungnam University College of Medicine, Daegu, Korea; 3 Department of Ophthalmology, School of Medicine, Kyungpook National University, Daegu, Korea; 4 Department of Ophthalmology, Kosin University college of medicine, Gospel Hospital, Busan, Korea; 5 J Eye Center, Busan, Korea; 6 College of Medicine, Pusan National University, Yangsan, Korea; 7 Institute of Health Sciences, Gyeongsang National University Hospital, Gyeongsang National University, Jinju, Korea; 8 Dong-A University Hospital, Busan, Korea; 9 Inje Univertisy, Haeundae Paik hospital, 875, Haeun-daero, Haeundae-gu, 48108 Busan, Korea; 10 Inje University Pusan Paik hospital, 875, Haeun-daero, Haeundae-gu, 48108 Busan, Korea

**Keywords:** Dexamethasone, Diabetic retinopathy, Exudate, Intravitreal injection, Macular edema

## Abstract

**Background:**

To investigate the effect of intravitreal dexamethasone implant (DEX implant) on hard exudate (HE) accompanying diabetic macular edema (DME).

**Methods:**

This study was a non-comparative non-randomized 1-year prospective interventional study. Patients with DME and HE were treated using DEX implant two or three times. Color fundus photography and optical coherence tomography (OCT) were performed at every visit. HE area was measured semi-automatically from the fundus photographs.

**Results:**

Thirty-five patients completed the study. Eleven patients (31.4%) received two injections, while the remaining received three times. HE area (primary outcome) significantly decreased from 1.404±2.094 mm2 (baseline) to 0.212±0.592 mm2 (last visit), which was 24% of the baseline HE area (*P*<0.001). HE1500 (HE within 1500 μm from the fovea) area also decreased significantly from 0.382±0.467 mm2 to 0.066±0.126 mm2 (*P*<0.001). Furthermore, anaverage best corrected visual acuity (BCVA) improvement of 4.4 Early Treatment Diabetic Retinopathy Study (ETDRS) letters was observed (from 49.9±18.3 to 54.3±20.4 letters) (*P*= 0.008). Central macular thickness (CMT) decreased from 455.8±23.6 μm to 366.8±31.1 μm (*P*=0.009). Repetitive measurements for entire study duration was analyzed using generalized estimating equations (GEE), where BCVA was related to age, CMT, and HE1500 area in multivariate analyses.

**Conclusion:**

DEX implant could reduce and suppress HE in DME for one year with two or three injections. And centrally located HE area (HE1500 area) is related to vision.

**Trial registration:**

ClinicalTrials.gov, NCT02399657, Registered 26 March 2015.

## Background

Diabetic macular edema (DME) is a major vision-threatening complication of diabetes mellitus. The overall prevalence of DME is estimated to be 6.96% in diabetic patients, with a cumulative incidence of approximately 25% in type 2 diabetes patients treated with insulin [1, 2]. DME has a poor prognosis if untreated, and eyes with DME involving the fovea showed moderate vision loss in 29% and visual recovery of 3 Early Treatment Diabetic Retinopathy Study (ETDRS) lines in only 5% of individuals [3]. Several clinical trials have demonstrated that intravitreal anti-vascular endothelial growth factor (anti-VEGF) injection reduces vision loss and even improves vision [4–6]. Intravitreal steroid injection has also been proved to be effective in DME treatment [7].

Hard exudate (HE) often accompanies DME. HE, which presents yellow-white deposits in retina, is thought to consist of lipid exudation. While HE can occasionally resolve spontaneously, it can form fibrotic lesions that lead to severe vision loss. HE is related to poor visual outcomes in patients with DME [8, 9]. Especially, subfoveal HE was demonstrated to be an independent risk factor for visual decline [10]. Several studies have shown HE resolution after using anti-VEGF or intravitreal dexamethasone (DEX) implant [9, 11, 12]. Some researchers showed that the steroid is superior to anti-VEGF agents [11]. In addition, DEX implant can be advantageous in that it stays for a longer duration in the eye. However, aforementioned studies had limitations regarding their short study durations and post hoc analyses in which HE was not the primary outcome of interest. Moreover, they showed conflicting outcomes about HE reduction. Therefore, we investigated the effect of DEX implant on HE in DME in a prospective cohort.

### Methods

Patients with DME and HE were recruited from 8 tertiary medical centers. DME was defined as macular thickening resulting from diabetic retinopathy and not by another cause. Cases were included if baseline central macular thickness (CMT) exceeded 300 µmand had visible HE within 1500 µm to the fovea. Additionally, patients were included if their vision ranged from 20/320 to 20/40. If both eyes were eligible, the eye with greater HE was selected. The main exclusion criteria were as follows: concurrent retinal disease that may provoke macular edema, intraocular surgery or intravitreal anti-VEGF injection within 3 months of study entry, intravitreal or subconjunctival steroid injection within 6 months of study entry, eyes with media opacity hindering imaging study required for study protocol, uncontrolled systemic disease, patients requiring systemic glucocorticoid or immunosuppressant treatment and patients not suitable for DEX implant. All study conduct adhered to the tenets of the Declaration of Helsinki, and the study protocol was approved by the Institutional Review Board.

All study eyes received DEX implant (Ozurdex; Allergan, Irvine, California, USA) right after screening evaluation (first injection) and at 4 or 5 month-visit (second injection). Additionally, from 8 to 11 months, the study eye could receive a third injection as to investigator’s determination based on the CMT exceeding 350 µm or exacerbation of vision more than 5 letters compared to best vision. Study visits were scheduled every month until 12 months following study initiation. Best corrected visual acuity (BCVA) measured using ETDRS chart, intraocular pressure (IOP), slit lamp examination, and fundus examination were performed every visit. Standard fundus photography and optical coherence tomography (OCT) (Cirrus OCT: Carl Zeiss Meditec, Dublin, CA or Spectralis OCT: Heidelberg Engineering, Heidelberg, Germany) were performed at every visit. Complete blood cell count, basic chemistry analysis including HbA1c and lipid profiles were measured at screening and final visit. Fluorescein angiography and indocyanine-green angiography were performed at baseline and final visit. Cataract was graded according to lens opacification classification system (LOCS III grading). Each investigator separately graded the cataract status.

**Fig. 1:**
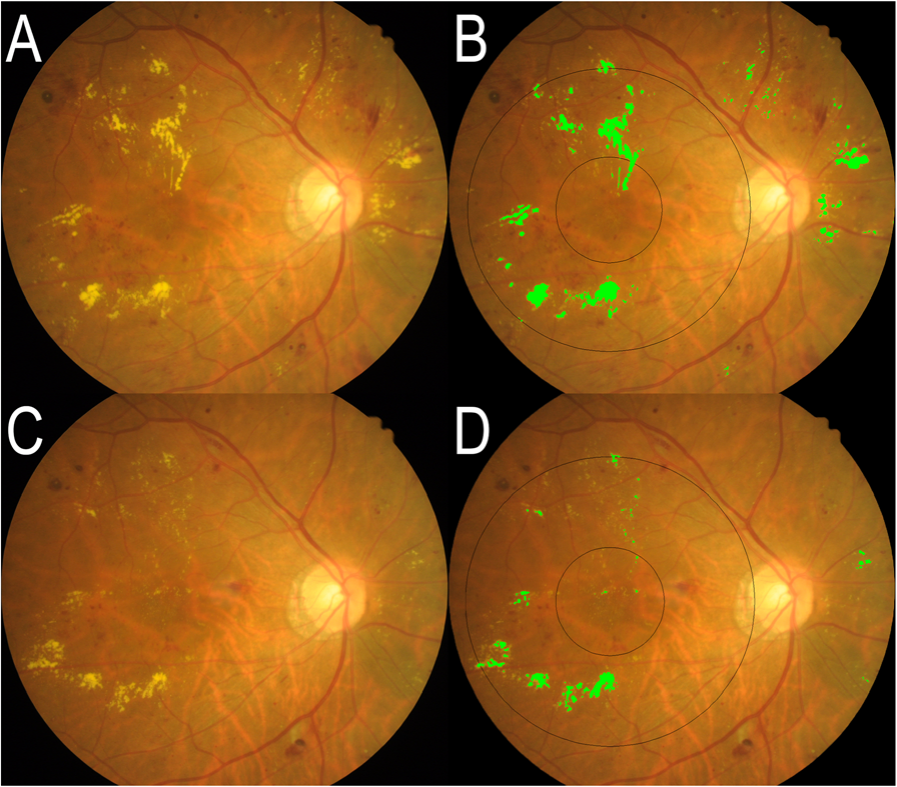
Fundus photograph and measurement of hard exudate in a representative case. **a** and **c** Fundus photograph of the same patient at baseline and last visit, respectively. **b** and **d** Hard exudate (HE) area analysis in **a** and **c**. Light green colored pixels indicate the identified HE. Vague tiny yellow particles are not marked as HE. Black circles are 3 mm and 8 mm diameter circles centered at the fovea. These circles are generated using superimposed optical coherence tomography fundus image as reference. HE area and HE1500 area are defined as the area of HE within 8 mm and 3 mm circle, respectively

Macular HE area within 1500 µm from the fovea center (HE1500 area) and within arcade (4000 µm from the fovea center, HE area) were measured from fundus photograph semi-automatically using ImageJ software (Rasband, W.S., ImageJ, U. S. National Institutes of Health, Bethesda, Maryland, USA). Two masked graders (GCJ and JHL) performed the analysis individually. Reference scale and fovea location were obtained from overlay of OCT image on fundus photo. OCT report image contains retinal image displaying scanned area and foveola. Thus, we are able to get actual scale of the fundus photo and foveola location by superimposing the OCT retina image on color fundus photo. After uploading fundus photography into ImageJ, green channel image was used for further processing. Basically, binary images separating HE were generated by the automatic threshold function of ImageJ and manual adjusting. In the binary image, pixels other than HE, including vessel or optic disc, was erased manually. Next, the total area of HE within 4000 µm and 1500 µm circle were measured (Fig. 1). Interclass correlation between the two graders was 0.95 (P < 0.001). The average value of HE area identified by the two graders was used for statistical analysis.

The presence of subfoveal fluid at initial examination was determined using OCT. Morphologic typing of macular edema as cystoid macular edema or diffuse retinal thickening followed the classification of Kim et al. [13]. Microaneurysm number was counted from fluorescein angiogram [14].

**Fig. 2:**
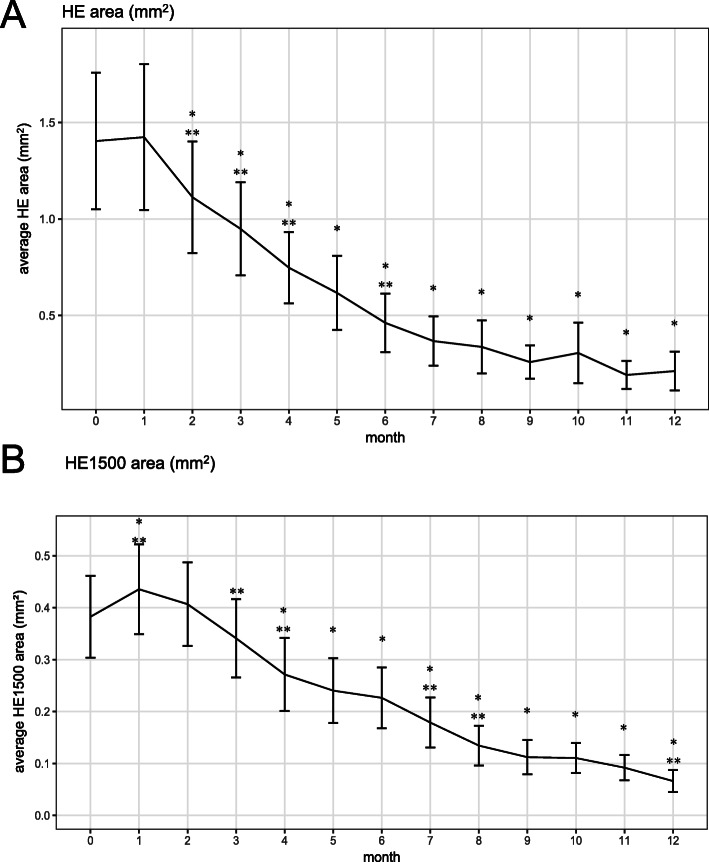
Line plots of hard exudate (HE) area. **a** Line plot of the average HE area of each visit. **b** Line plot of the average HE1500 area. HE1500: HE within 1500 µm from the fovea. * *p* < 0.05, paired t-test compared to baseline value, ** *p* < 0.05, paired t-test compared to value of 1 month ago

**Fig. 3:**
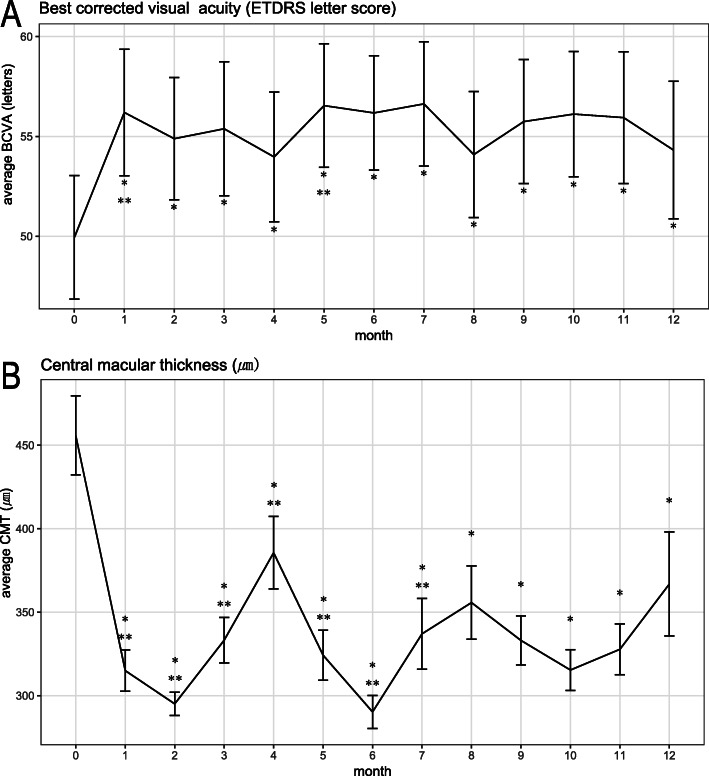
Line plots of average BCVA and CMT (**a**) Line plot of the average best corrected visual acuity (BCVA) measured by Early Treatment Diabetic Retinopathy Study (ETDRS) chart. **b** Line plot of the average central macular thickness (CMT). * *p* < 0.05, paired t-test compared to baseline value, ** *p* < 0.05, paired t-test compared to value of 1 month ago

Paired t-tests were used to compare the change of variables from the initial value at each time point. (Table 1; Figs. 2 and 3) Linear regression analysis was used to determine the factors that influenced initial vision and HE area. HE area was log-transformed to be normalized in linear regression analysis. For analyzing the repetitive measurements, generalized estimating equation (GEE) models were generated for univariable and multivariable analyses to estimate the relationships of outcome variables with age, sex, routine laboratory data, cataract progression, event of increased IOP and the number of Dex injections. Potential relating variables with a P value of ≤ 0.10 on univariate analysis were tested on multivariable analysis. An autoregressive correlation structure was assumed in these models to account for the within subject correlation. Statistical analyses were performed using the software package SPSS 23.0 (IBM Corp. Released 2015. IBM SPSS Statistics for Windows, Version 23.0. Armonk, NY: IBM Corp.). A P value of < 0.05 was considered statistically significant. Sample size was determined as 48 based on following parameters. Proportion of patients who showed reduction of HE was estimated to be 0.9, significance level was 0.05, margin of error was 0.1 and drop-out rate was 0.25.

The study protocol followed the guidelines of the Declaration of Helsinki. Written informed consent was obtained from all of the participants before the study began.

## Results

**Fig. 4:**
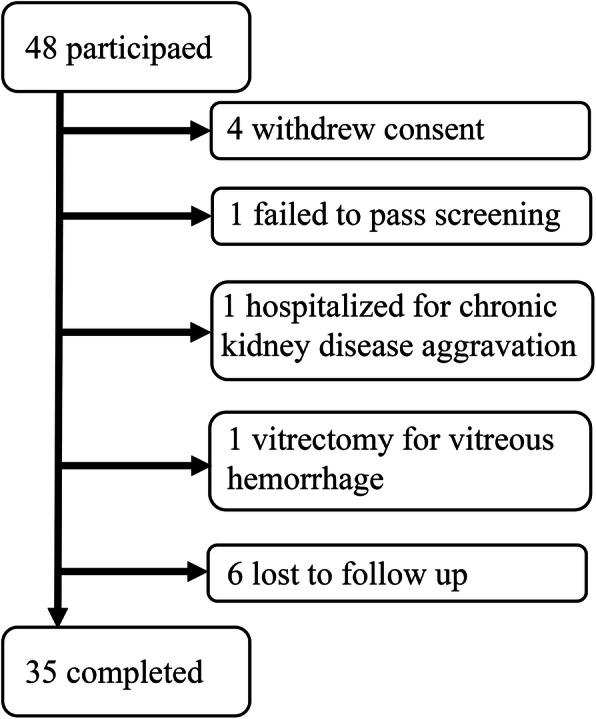
Flowchart of study participants Flow chart describes the numbers of participants who had initially enrolled and have withdrawn during the study

Forty-eight patients were initially enrolled. Four patients withdrew consent, one patient failed to pass screening, one patient underwent vitrectomy due to vitreous hemorrhage, one patient was hospitalized for chronic kidney disease aggravation and six patients were lost during follow up. Finally, 35 patients completed the study protocol. (Fig. 4) The two required injections were done in all the participants and additional third injection was done in 24 patients (68.6%) depending on investigator’s decision. The mean age was 59.3 years (range 29 to 73, standard deviation (SD) 9.8) and 12 (34.3%) were male. Although study protocol allowed for laser treatment as a rescue therapy after 4 months from first injection, no patients underwent laser treatment. Among blood chemistry analysis, low density lipoprotein (LDL) cholesterol decreased significantly after one year, and microaneurysm count located within 3000 µm of the fovea decreased. Detailed baseline characteristics and blood chemistry are presented in Table 1.

**Table 1:** Characteristics of study populations

	Baseline values	After 1 year values	P*
Age (Avg [SD])^†^, years	59.3 (9.9)		
Male: Female	12:23		
Hypertension	12 (34.3%)		
CKD	2 (5.6%)		
Dyslipidemia	7 (19.4%)		
HbA1c (Avg [SD])	7.96 (1.61) %	7.56 (1.22) %	0.105
BUN (Avg [SD])	18.1 (5.2) mg/dl	20.4 (7.6) mg/dl	0.052
Cr (Avg [SD])	1.02 (1.19) mg/dl	1.19 (0.99) mg/dl	0.073
TG (Avg [SD])	171.0 (77.2) mg/dl	148.9 (82.6) mg/dl	0.122
Total cholesterol (Avg [SD])	183.8 (53.5) mg/dl	170.4 (39.0) mg/dl	0.222
HDL (Avg [SD])	50.0 (15.0) mg/dl	50.7 (12.0) mg/dl	0.859
LDL (Avg [SD])	120.4 (52.0) mg/dl	100.8 (37.1) mg/dl	0.048^‡^
diffuse: focal edema	16 (45%):19 (55%)		
Presence of SRF	11 (31%)		
Microaneurysm count (Avg [SD])	15.3 (13.3)	13.1 (10.5)	0.032^‡^

*CKD* chronic kidney disease, *Presence of SRF* presence of macular subretinal fluid, *BUN* blood urea nitrogen, *Cr* creatinine, *TG* triglyceride, *HDL* high density lipoprotein, *LDL* low density lipoprotein

*paired t-test; †Avg [SD]: average [standard deviation]; ‡*P* < 0.05

Average HE area (SD) decreased from 1.403 (2.094) mm^2^ at baseline to 0.211 (0.592) mm^2^ at final visit (*P* < 0.001, paired t-test), which was 26% of the total baseline area. Mean HE1500 area (SD) also decreased significantly from 0.382 (0.467) mm^2^ at baseline to 0.066 (0.126) mm^2^ at final visit (*P* < 0.001, paired t-test), a decrease to 17% of the initial total HE1500 area. Among the 33 patients who had an HE1500 larger than 0.001 mm^2^ measured by photo analysis at baseline, HE1500 area decreased in 26 (79%) patients at 10 months and in 28 (85%) by final visit (Fig. 2). HE area significantly decreased from month 2 and HE1500 area decreased from month 3. HE1500 area increased significantly at month 1. (0.382 mm^2^ to 0.435 mm^2^, *P* = 0.009)

The improvement of average BCVA (SD) was 4.4 (9.3) letters (average BCVA [SD] were 49.9 [18.3] letters at baseline and 54.3 [20.3] letters at 1 year [*P* = 0.008, paired t-test]). Average CMT (SD) decreased significantly from 455.8 (23.6) µm at baseline to 366.8 (31.1) µm at 1 year (*P* = 0.009, paired t-test) (Fig. 3). The reduction of average CMT (SD) was 89 (191.5) µm. (Fig. 3) The proportion of patients whose CMT was under 290 µm was 34.2% (12 out of 35) at last visit. HE area reduction (Initial HE area – final HE area) showed positive correlation with BCVA improvement (Final BCVA – Initial BCVA) (Pearson correlation coefficient = 0.161, *P* < 0.001). However, there was no correlation between HE and CMT reduction.

Baseline BCVA was related to initial HbA1c. Baseline HE area was related to total cholesterol, LDL level, and microaneurysm count in the univariable analysis. Microaneurysm count was only significantly correlated with HE area in the multivariable analysis. (Table 2)

**Table 2:** Univariate and multivariate analysis of factors affecting baseline HE and visual acuity

	Baseline BCVA^a^		Baseline HE area^b^		
Variable	Beta	*P* value	Beta	*P* value	
		Univariable		Univariable	Multivariable
Male	3.098	0.631	0.915	0.127	
Presence of SRF	-4.8	0.476	0.169	0.773	
Diffuse type ME	-6.09	0.319	-0.077	0.708	
Age	-0.33	0.288	0.009	0.708	
HbA1c	-4.191	0.02*	-0.077	0.746	
BUN	-0.755	0.203	0.007	0.874	
Cr	3.856	0.445	0.284	0.209	
TG	0.011	0.794	0.003	0.19	
Total cholesterol	0.006	0.918	0.009	0.001*	0.165
HDL	-0.018	0.933	0	0.991	
LDL	0.077	0.506	0.008	0.013*	0.72
Microaneurysm count	-0.353	0.116	0.036	< 0.001*	< 0.001*
HE area	-0.171	0.908			
HE1500 area	-6.839	0.316			
CMT	-0.025	0.258	0.002	0.189	

*BCVA* best corrected visual acuity, *Presence of SRF* presence of macular subretinal fluid, *DME* diabetic macular edema, *BUN* blood urea nitrogen, *Cr* creatinine, *TG* triglyceride, *HDL* high density lipoprotein, *LDL* low density lipoprotein, *HE* hard exudate, *HE1500* HE within 1500 μm from the fovea, *CMT* central macular thickness

*P value less than 0.05; ^a^linear regression analysis; ^b^linear regression analysis after log transformation of HE area

The generalized estimating equation (GEE) was generated where repetitive measurements of HE area or BCVA in one person over 1 year were designated as outcome variables and visit month was a within variable. When BCVA was outcome variable, initial HbA1c, initial microaneurysm count, age and repetitive measurements of CMT, HE area, HE1500 area were significantly correlated to BCVA. In multivariable model, age, HE1500 area and CMT were statistically meaningful parameters (Table 3). However, when HE area was assigned as outcome variable, neither parameters in Table 3 showed any statistical relationships with HE area. (HE area and HE 1500 area were omitted in this analysis).

**Table 3:** Factors affecting repetitive measurements of BCVA^a^ for 1 year

Variable	Beta	*P* value	
		Univariable^b^	Multivariable^b^
Male	4.824	0.47	
Presence of SRF	-8.378	0.174	
Diffuse type DME	-2.128	0.729	
Age	-0.594	0.038*	0.011*
HbA1c	-3.842	0.035*	0.164
BUN	-0.583	0.445	
Cr	4.354	0.142	
TG	-0.011	0.75	
Total cholesterol	0.01	0.866	
HDL	0.055	0.768	
LDL	0.061	0.502	
Microaneurysm count	-0.382	0.014*	0.201
IOP increase (> 25 mmHg)	0.212	0.758	
Cataract progression	-0.321	0.132	
HE area^a^	-0.807	0.383	
HE1500 area^a^	-4.457	< 0.001*	0.004*
CMT^a^	-0.042	0.001*	< 0.001*

*BCVA* best corrected visual acuity, *Presence of SRF* presence of macular subretinal fluid, *DME* diabetic macular edema, *BUN* blood urea nitrogen, *Cr* creatinine, *TG* triglyceride, *HDL* high density lipoprotein, *LDL* low density lipoprotein, *IOP* intraocular pressure, *HE* hard exudate, *HE1500* HE within 1500 µm from the fovea, *CMT* central macular thickness

*P value less than 0.05

^a^BCVA, HE area and HE1500 area indicates the values observed in every visit of all the study participants. Because they were clustered data of the same person, generalized estimating equation (GEE) was used as statistical analysis

^b^The outcome variable was BCVA. The within-subject variable was the visit month. An autoregressive correlation was used as a working correlation

Cataract progression was observed in 16 out of 35 (45.7%) patients. LOCS grading revealed aggravated nuclear opacity (37.1%) and post-subcapsular opacity (20.0%). Two patients underwent cataract surgery during the study duration. IOP exceeding 25mmHg at any time was observed in five patients (11%) and was controlled through the application of topical anti-glaucoma medication. Only one patient recorded 31mmHg once, and the pressure was controlled thereafter. One patient was hospitalized for chronic kidney disease aggravation and dropped out of the study. Other adverse events (AE) included eye discharge, subconjunctival hemorrhage, burning sensation of eye, upper respiratory infection symptom, vitreous hemorrhage, high blood pressure, gastrointestinal trouble, gastroenteritis, and tinnitus. Any progression of cataract or event of increased IOP (over 25mmHg) have not affected visual acuity. (Table 3)

## Discussion

This study showed that HE in DME decreased significantly during a year using DEX implant injection twice or three times over the study period. To the best of our knowledge, this is the first prospective interventional study focused on HE in DME using DEX implant for 12 months. HE area and centrally-located HE1500 area decreased continuously. Moreover, HE1500 was closely related to visual acuity. A distinct feature of HE area compared to other outcomes is its continuous regressing pattern except for the initial increase or plateau. CMT decreased after injection and reached its lowest level around 2 months after injection, at which point CMT increased until the next injection. BCVA also showed maximal improvement around 2 months after injection and then decreased. Therefore, average line plots resemble a U- or inverted U-shape for CMT and BCVA, respectively (Fig. 3). This pharmacokinetic effect of DEX implant on CMT and BCVA is consistently reported in other studies [7]. On the other hand, the current study revealed a continuous pattern of HE area regression. Other studies using DEX implants, which had shorter study durations or longer visit intervals than the current study, have not shown such a continuous reduction of HE [11, 12]. HE area and HE1500 area showed increase at 1 month after first injection (p value = 0.7358 and 0.009, respectively). This does not match to the most rapid improvement of vision and CMT at this time point. We don’t know whether this is real increase of HE because we were not able to measure the total volume through fundus photo. Abrupt resolution of macular edema and relatively lagged absorption of HE could make HE distinctively visible and this might result in increase of HE area measured using fundus photo segmentation. Relative faster resolution of fluid in central than peripheral macula might explain only significant increase of HE1500 at 1 month. This phenomenon of HE increase at early periode is also reported in monthly ranibizumab injection study [15].

The current study does not have a control group not having any intervention or undertaking different treatment. Therefore, we should be cautious when interpreting whether HE resolution is a substantial effect of DEX implant. There are several studies investigating the efficacy of intravitreal injection for the reduction of HE in DME. Secondary analysis of the BEVORDEX study investigated the efficacy of dexamethasone implant or bevacizumab on the regression of HE [11]. Both DEX implant and bevacizumab are effective at reducing HE. However, this study suggested that DEX implant provided more rapid regression of HE from the foveal center than bevacizumab at 12 months. Another post hoc analysis was performed using the “RISE and RIDE study” [9]. This study reported that monthly ranibizumab injection resulted in significant reduction of HE area compared to the sham group. Total resolution of HE was observed in 60% of the ranibizumab group and 36% in the sham group. There are several studies which have conducted short term observation of HE after intravitreal injection. Monthly bevacizumab injection has been shown to not reduce HE area within 6 months [16]. In addition, HE count increased after three loading injections and an additional single treatment of anti-VEGF for 6 months in a separate study [17]. DEX implant and triamcinolone injection have been shown to reduce HE, but not bevacizumab, in a 3-month observational study [12]. To summarize these studies, monthly ranizibumab was found to be superior to a “laser treatment as needed” group over a 2-year period. Monthly bevacizumab was as effective as DEX implant every 16 weeks after 2 years [11]. Specifically, DEX implant reduced HE in a short period even when the efficacy of anti-VEGF was controversial.

DEX implant is advantageous due to its injection schedule, which is relevant to economic burden and time consumption. Ranibizumab or bevacizumab were injected with fixed monthly dosing in studies suggesting efficacy on HE. However, monthly injection is not practical in ordinary clinical circumstances. Real world observational studies report 4 to 7 anti-VEGF injections per year, which is much less than monthly dosing of pivotal studies [18, 19]. However, the average 2.7 DEX implant at 1 year observed in the present study is consistent with 2.4 injection per year in usual practice [20]. Thus, DEX implant for DME with HE is economically feasible and an effective method even though monthly anti-VEGF is equally effective.

The current study also showed anatomical and visual improvements. The average CMT reduction after 1 year was 89 µm. This reduction was less than the 187 µm decrease in the BEVORDEX study which had a similar injection schedule as that of the current study; the result was rather comparable with 60 µm reduction in the real world study [21, 22]. Differences in the study population can explain these discrepancies. Present study requires macular HE and is likely to include more proportion of treatment resistant patients. GEE analysis showed that HE area was related to CMT (β = 21.2, P = 0.002). HE was also strongly correlated with CMT reduction in DRCR protocol I [23]. The average visual gain was 4.4 letters, and 35% of patients had a visual gain of > 10 letters. This visual gain is comparable with the BEVORDEX study reporting 5.6 letters and 40%, respectively [24]. Another important factor explaining current CMT and BCVA outcomes can be the elapsed time after final injection. As our last observation was performed after passing the peak efficacy of DEX implant and a third injection was performed only in 68%, CMT and VA outcome could be underestimated at the final visit.

We could not find factors associated with changes in HE, although there were several factors relevant to baseline HE and BCVA. Baseline HE area was related to total cholesterol and LDL concentrations. This is consistent with the results of previous reports describing that higher total cholesterol and LDL cholesterol concentrations were correlated with retinal HE [25, 26]. Baseline BCVA was correlated with lower initial HbA1c level in current study. HbA1c reflects long-term glycemic control and intensive glycemic control delays the progression of diabetic retinopathy [27]. HbA1c levels was also correlated with baseline hyperreflective foci count on OCT [28]. There is conflicting evidence of HbA1c influence on visual response to anti-VEGF treatment. HbA1c was not associated with BCVA change in ‘RISE and RIDE’ study, while it was associated with visual acuity improvement in ‘VISTA and VIVID trials’ and ‘Protocol T’ [29–31].

HE regressed regardless of other parameters in the current study. However, low HbA1c and low cholesterol levels showed a beneficial effect on HE reduction using the anti-VEGF treatment [17]. We may presume that the efficacy of the DEX implant on HE reduction is strong enough to overcome the influence of other factors. Or we could not find any contributing factors due to small study population.

We found that HE area was related to BCVA. In our study, baseline HE was not relevant to BCVA whether it was located centrally or not. Centrally-located HE area (HE1500) was related to BCVA in both univariable and multivariable GEE analysis, as well as CMT. HE area reduction showed weak positive correlation with BCVA improvement. The predictive value of HE on visual outcome is controversial. In the ETDRS study, after adjusting for other factors, foveal HE was an independent risk factor for worse VA outcome [8]. Sadda et al. showed that baseline foveal HE were associated with worse visual outcomes [15]. BEVORDEX and RISE RIDE post hoc analysis showed that HE does not affect visual outcome. DRCR.net protocol I revealed that patients having baseline HE showed better VA outcome [23]. It is not clear whether HE reduction is the reason of better visual outcome or not in protocol I. Unlike CMT which directly affected central vision, the impact of HE on vision might be limited or indirect. Older age was negatively correlated to vision in current study. Data from the RESTORE study revealed that larger treatment benefits of VA were associated with younger age,[23] suggesting that younger patients have better visual outcomes.

Corticosteroid relieves the abnormal inflammatory process and lowers VEGF level that may stabilize the barrier function of vessels [32]. Moreover, corticosteroid can stabilize the blood retinal barrier by regulating endothelial junction proteins or water channel in Muller glial cells [33, 34]. Thus, corticosteroid might ameliorate the pathologic condition of macromolecule leakage from vessels and maintain homeostasis to help the removal of HE. Additional anti-inflammatory effects of corticosteroid might result in a faster reduction of HE compared to anti-VEGF agents.

Cataract progression and increased IOP are important AE of DEX implant. In the current study, we experienced 47% of patients showing cataract progression and two patients underwent cataract surgery. IOP exceeding 25 mmHg at any time was observed in five patients (11%) and this was relatively small portion comparing 35% in MEAD study [7]. Except for cataract progression and increased IOP, AEs reported in this study have low clinical relevance with study drug use. In current study, cataract progression was not related to vision. It might be based upon the relative small study population or finding that cataract generally appears in the second year after intravitreal steroid therapy [35].

The present study has several limitations. First, we don’t have control group to compare the effect of the study drug. Although we can infer the effect of the intervention by comparing to previous reports using other drugs, natural course data of HE in DME is limited. In addition, our study population was comprised of a relatively small number of patients, and therefore, it may not be large enough to reveal the factors influencing the HE reduction. However, the current study accomplished the study objective of investigating HE area change in a prospective nature.

This study clearly showed that HE in DME regressed continuously using the DEX implant except right after the first injection. We also observed visual gain and CMT reduction, which are consistent with other studies. Majority of previous studies have reported that steroid is superior in reducing HE than anti-VEGF agents within a short duration of approximately 1 year. Moreover, DEX implant schedule in the current study was an amenable treatment interval close to that in the routine clinical practice. Centrally-located HE near the fovea is related to vision. Thus, DEX implant can be considered as a proper treatment option, especially when DME is accompanied by foveal HE.

## Conclusions

Dex implant was able to reduce and suppress HE of DME in generally continuous and rapid manner by injecting two or three times for a year. Dex implant improved vision and ameliorate macular edema as well. Moreover, centrally located HE around fovea was related to visual acuity. Thus, Dex implant can be a beneficial treatment option when DME accompanied by HE.

## Data Availability

The data that support the findings of this study are available from the corresponding author, [H.W.K.], upon reasonable request.

## Abbreviations

DEX implant: Intravitreal dexamethasone implant
HE: Hard exudate
DME: Diabetic macular edema
OCT: Optical coherence tomography
BCVA: Best corrected visual acuity
ETDRS: Early Treatment Diabetic Retinopathy Study
CMT: Central macular thickness
HE1500 area: Centrally located HE area
anti-VEGF: Anti-vascular endothelial growth factor
IOP: Intraocular pressure
GEE: Generalized estimating equation
SD: Standard deviation
LDL: Low density lipoprotein

## CONSORT guidelines

This study adheres to CONSORT guidelines
